# Readability of patient information and consent documents in rheumatological studies

**DOI:** 10.1186/s12910-016-0126-0

**Published:** 2016-07-16

**Authors:** Bente Hamnes, Yvonne van Eijk-Hustings, Jette Primdahl

**Affiliations:** Hospital for Rheumatic Diseases, Margrethe Grundvigs vei 6, 2609 Lillehammer, Norway; Department of Patient & Care, Maastricht University Medical Centre, PO Box 5800, 6202 AZ Maastricht, The Netherlands; Institute for Regional Health Research, University of Southern Denmark, Winsløwparken 19, 5000 Odense, Denmark; Hospital of Southern Denmark, Kresten Philipsens vej 15, 6200 Aabenraa, Denmark; King Christian 10th Hospital for Rheumatic Diseases, Toldbodgade 3, 6300 Graasten, Denmark

**Keywords:** Informed consent document, Readability, Gunning's Fog Index, Simple Measure of Gobbledygook

## Abstract

**Background:**

Before participation in medical research an informed consent must be obtained. This study investigates whether the readability of patient information and consent documents (PICDs) corresponds to the average educational level of participants in rheumatological studies in the Netherlands, Denmark, and Norway.

**Methods:**

24 PICDs from studies were collected and readability was assessed independently using the Gunning’s Fog Index (FOG) and Simple Measure of Gobbledygook (SMOG) grading.

**Results:**

The mean score for the FOG and SMOG grades were 14.2 (9.0–19.0) and 14.2 (12–17) respectively. The mean FOG and SMOG grades were 12.7 and 13.3 in the Dutch studies, 15.0 and 14.9 in the Danish studies, and 14.6 and 14.3 in the Norwegian studies, respectively. Out of the 2865 participants, more than 57 % had a lower educational level than the highest readability score calculated in the individual study.

**Conclusions:**

As the readability level of the PICDs did not match the participants’ educational level, consent may not have been valid, as the participants may have had a limited understanding of what they agreed to participate in. There should be more focus on the readability of PICDs. National guidelines for how to write clear and unambiguous PICDs in simple and easily understandable language could increase the focus on the readability of PICD.

**Electronic supplementary material:**

The online version of this article (doi:10.1186/s12910-016-0126-0) contains supplementary material, which is available to authorized users.

## Background

This study examines the readability of written patient information and consent documents (PICDs) used in rheumatology research. Ethical guidelines for medical research on human beings, which includes PICDs, were developed by the World Medical Association in Helsinki in 1964 [1]. The guidelines has been revised several times, the last time in 2013 [2]. The guidelines have also been implemented by National Ethic Committees [3–5].

Participants in medical research must give their informed consent before they can participate in a study. The consent must be free and informed, which means that the person should not experience any kind of pressure and should have the necessary information about the research which is to be conducted. This comprises knowledge about the purpose of the research project, duration, methods and procedures, types of expected results, and planned dissemination. Additionally, participants should have knowledge about the study’s potential benefits and risks, or negative consequences such as discomfort. Finally, participants should also receive information about confidentiality and data handling, in addition to knowledge about, that they can withdraw from the study at any time without any explanation and without influence on their clinical care [2]. The information should be written and easy to understand and carry as little risk as possible of misunderstanding. In medical research on humans, PICDs are key requirements, which must be approved by ethical committees [3–5]. In 2012, the European Commission described the literacy level in the population as important. Poor literacy is a hidden problem in most European societies, where one in five young people and adults lacks basic literacy skills [6]. Health literacy means the ability to read and understand basic health information, make appropriate health decisions, and take appropriate actions accordingly [7, 8]. People with poor health literacy will probably not understand a PICD if the text is too complex. In addition to the complexity of the text, participants’ reading ability is an important factor in ensuring their understanding of the written information. This must be taken into account when developing written materials like PICDs to ensure that patients are provided understandable information about the research study. This will allow them to make an informed decision about study participation [9].

Readability describes the number of years of schooling necessary to read and understand a text and can be measured by standardized instruments. Gunning’s Fog Index (FOG) [10] and the Simple Measure of Gobbledygook (SMOG) [11] are among the most commonly used instruments [12]. SMOG has a high correlation with FOG (0.95–0.99) [13, 14]. Scores from the two indexes reflect the average years of schooling a person needs to be able to read and understand a text. A SMOG or FOG score of 5 is equal to 5 years of schooling. In the health literature, the recommended readability level ranges from grades 5 to 9 [15–17].

Often PICDs have higher readability level than the study population or recommended readability level [15, 16, 18, 19]. In a study with 40 PICDs in anaesthesiology research, the readability score for the FOG was 11.9. This score was significantly higher than the average literacy level of the Australian and New Zealand populations, where 44–46 % people have a literacy level below the required minimum to meet the complex demands of everyday life [16]. In a Croatian study a readability score of 13.25 was found in informed consent documents in diagnostic and therapeutic procedures where 80 % of the population older than 15 years had less education [19]. Another study of patient information leaflets on physiotherapy showed that only 11 out of 33 leaflets (33 %) met the recommended readability level of grade 9 [15].

In addition to the readability level, the content of the text are essential for understanding. Studies on how participants understand medical consent documents showed four sources of uncertainty: language, information about risks and hazards, the nature of the procedure, and the documents’ composition and format [20]. This is congruent with the perspective of research ethical committee members which concluded that language, structure and format of the patient information sheet should be improved [21].

A review of interventions used to improve participants’ understanding of informed consent in research showed that one-to-one discussion and participant feedback with a study team member improved the participant’s understanding of the PICD. Other interventions such as multi-media and enhanced PICDs showed mixed levels of improvement in participant understanding [22]. Latest reviews from 2012–14 on improving participants understanding of PICDs, showed that enhanced PICDs and extended discussions were most effective [23]. For older participants interactive multimedia and written materials which were easy-to-read, increased the patients understanding of the consenting procedure [24]. For people with low literacy the most effective strategy was talking one-to-one with a study member, but this result is based on a single study only [25].

In the literature, it is recommended to conduct readability tests on patient materials, but this is not yet common practice [26]. During the European League Against Rheumatism Congress in 2010 a workshop regarding readability stressed the importance of the topic and it was recommended to be followed up. This study aims to document whether the readability of PICDs corresponds with reported education or schooling level of the participants enrolled in rheumatological studies.

## Methods

### Materials

We identified relevant clinical studies in the rheumatology field from each of the authors’ home countries (the Netherlands, Denmark, and Norway). Studies were selected in the researchers’ own languages to ensure that at least one with the native language reviewed all consent documents in each language.

An initial search was performed in PubMed in July 2014: #1: rheumato* OR arthritis OR arthro* OR psoriatic OR ankylosing spondylitis OR Bechterew OR lupus OR fibromyal* OR (inflammatory AND joint) OR chronic widespread pain #2: (Norway OR Norwegian; Holland OR Dutch; Denmark OR Danish) #1 AND #2. Additionally, we used filters to identify clinical trials that were performed within the last 10 years on humans; we searched for trials whose full texts were available. To include enough Danish rheumatological studies, we repeated the search for Danish studies in Cumulative Index to Nursing and Allied Health Literature (CINAHL).

Inclusion criteria were studies published during the past 10 years (2004–2014), study population ≥ 18 years of age, and a patient population with a specific rheumatic disease. Exclusion criteria were register-based studies, literature reviews, or other studies where no patient information or consent forms were used, studies from other countries than the Netherlands, Denmark, and Norway, and studies where no information about educational level (i.e. years of education, literacy level, and other proxies) was reported.

The titles and abstracts of the identified studies were screened according to the admission criteria. If a study was not excluded based on the title or the abstract, the full text was retrieved and assessed according to the admission criteria. All authors screened the retrieved titles, abstracts, and full text from their own countries.

The first authors of the included studies were contacted through e-mail. We contacted the authors of the most recent studies first. They were informed about the aims of our study and were asked whether they would share the patient information and consent forms used in their study. If the first author did not respond within a week, we contacted the last author who was considered to have overall responsibility for the study. For one Danish and one Norwegian study, the authors were contacted for information about participants’ education level, which was not stated in the study, although the authors knew it was collected [27, 28].

As we expected each author could possibly reuse parts of the text from previous PICDs, we included only one study from each first author unless we knew that different people had been responsible for the development of the PICD. When data from the same study was reported in several papers by different first authors, only the author from the most recent paper was contacted. We aimed to be able to retrieve patient information and consent forms from 10 studies from each country and included studies that fulfilled the selection criteria consecutively.

### Analysis

To assess the readability of the material, we applied the FOG [10] and SMOG [11] to each set of PICD. FOG grading was calculated based on a sample of approximately 100 words. The average number of words per sentence was calculated by dividing the number of words by the number of sentences in the sample. Words with three or more syllables were considered hard words. The number of hard words were counted. If polysyllabic words were repeated, only the first instance was counted. Three-syllable words made up of a two-syllable word with endings like -e, −er, −es, and -ing were omitted. The summed number of words per sentence and hard words were then multiplied with 0.4, and this gave the final score for each sample of approximately 100 words. We used the first 100 words and last 100 words of the text for scoring FOG 1 and FOG 2. SMOG grading was performed on 10 consecutive sentences at the beginning, 10 in the middle, and 10 near the end of the text. The number of words with three or more syllables was counted (including repetitions). The square root of the number of polysyllabic words (of the nearest perfect square) was calculated and the number ‘3’ was added to reach the final score [11]. In case the text did not contain 30 sentences for the SMOG grading, we assessed 10 sentences from the beginning of the text, the following 10 sentences, and finally 10 sentences counted backwards from the end of the text. Thus, some sentences were included twice.

In case abbreviations were defined, the abbreviated version was not counted as a hard word no matter the number of syllables. Headings and subheadings in the text were calculated as sentences only in case they consisted of at least two words.

Each PICD was graded independently by hand by two of the authors. Each author scored all the PICDs from her own country and half of the PICDs from each of the two other countries. Next, the grades were compared. In case of discrepancy between the calculated score, the third author scored the material as well, and a final grading was reached by comparison and discussion.

Next, we compared the participants’ educational or schooling level with the readability grade of the PICD for each single study. Since the age for starting school and the educational system varies from country to country, the years of schooling were reported based on each country’s system (Table 1). The proportion and number of participants with an educational or schooling level lower than the highest score for the FOG or the SMOG were reported.

**Table 1:** Gunning’s Fog Index (FOG) and Simple Measure of Gobbledygook (SMOG) grade levels of studies and participants’ educational levels

Study no	Study design	No of participants (*n*)	Mean age and sex	Participants years of schooling or education level	FOG grade 1	FOG grade 2	SMOG grade	Participants with lower educational level than required to understand the PICD % (n)
The Netherlands								
1 [47]	Evaluation of self-management programs	19	21 years (*n* = 9) and 22 years (*n* = 10), female 84 %	Low 1^a^ Middle 13^b^ High 5^c^	11.0	9.4	13	5 % (1)
2 [48]	Cross-sectional study	333	47 years, female 100 %	Low 15^d^ Middle 256^e^ High 62^f^	10.1	9.0	13	5 % (15)
3 [49]	Randomised controlled study	199	62 years, female 65 %	Low 28^d^ Middle 79^e^ High 91^f^ Missing 1	11.7	15.5	13	54 % (107)
4 [50]	Randomised controlled study	82	48 years, female 92 %	Low/intermediate 50 High 32	15.6	11.1	13	61 % (50)
5 [51]	Randomised controlled study	203	In five groups: 39 to 43 years, female 92–100 %	Low 108 Middle 68 High 27	12.3	17.6	14	87 % (176)
6 [52]	Qualitative focus group study	20	57 years, female 75 %	Low 3^d^ Middle 12^e^ High 5^f^	13.8	11.8	12	75 % (15)
7 [53]	Randomised controlled study	158	47 years, female 94 %	Primary (mean 7 years) 8 Secondary (mean 12 years) 128 Tertiary (mean 17 years) 22	15.6	13.9	15	86 % (136)
Denmark								
1 [54]	Randomised crossover study	20	67 years, female 100 %	Elementary school 5 High school 0 <4 years at University level 9 4–6 years at University level 2 >6 years at University level 4	16.5	12.9	14	70 % (14)
2 [55]	A prospective study	315	55 years, female 77 %	None 67 Vocational 102 Higher 146	18.7	16.8	16	54 % (169)
3 [56]	A qualitative interview study	16	50 years, female 75 %	No formal education 2 Minimal education 7 Secondary education 4 University 3	13.0	11.0	13	69 % (9)
4 [57]	A qualitative interview study	13	44 years, male 100 %	Minimal education 1 Secondary education 9 University 3	11.5	17.0	15	77 % (10)
5 [58]	Randomised controlled study	287	63 years, female 87 %	<11 years 232 ≥11 years 55	13.6	17.9	15	81 % (232)
6 [59]	Qualitative focus group study	32	58 years, female 59 %	Unskilled 3 Vocational 18 Undergraduate 6 Graduate 5	15.2	13.8	14	84 % (27)
7 [28]	A qualitative interview study	31	38–89 years, female 85 %	<10 years 4 10–12 years 7 > 12 years 20	17.8	14.4	17	36 % (11)
Norway								
1 [60]	Randomised controlled study	57	Group 1: 68 and Group 2: 69 years, female 56 %	≤12 years 24 > 12 years 33	16.9	11.4	13	42 % (24)
2 [61]	Randomised controlled study	141	58 years, female 69 %	≤12 years 88 > 12 years 53	19.0	15.1	15	63 % (88)
3 [62]	Randomised controlled study	95	49 years, female 35 %	≤12 years 58 > 12 years 37	16.6	13.8	15	61 % (58)
4 [63]	Comparing treatment	153	Group 1: 57 years, female 89 %. Group 2: 51 years, female 53 %	≤12 years 87 > 12 years 65	15.2	18.7	14	57 % (87)
5 [64]	Randomised controlled study	68	50 years, female 68 %	Elementary school 13 High school 36 College/university 19	13.3	13.6	14	72 % (49)
6 [65]	Randomised controlled study	135	44 years, all female	≤10 years 21 11–13 years 49 > 13 years 53 Unknown 12	14.9	9.8	14	52 % (70)
7 [66]	Follow up study	134	55 years, female 87 %	9 years 36 ≤ 12 years 43 > 12 years 55	12.8	13.3	14	59 % (79)
8 [27]	A longitudinal study	281	46 years, female 58 %	≤9 years 38 10–12 years 118 > 12 years 125	12.8	13.7	15	56 % (156)
9 [29]	Randomised controlled study	107	M-group 48 years, N-group 51ys, female 43 %	Mean years of education 13 years (M-group) 12 years (N-group)	15.0	18.7	15	Cannot be calculated
10 [67]	Randomised controlled study	73	54 years, female 79 %	≤12 years 36 > 12 years 37	12.9	13.6	14	49 % (36)
Participants in 23 studies		2865^g^						57 % (1619^g^)

Explanation of years of schooling or education level (a–f)

^a^Vocational training

^b^Advanced vocational training

^c^College/University training

^d^Primary school/lower vocational secondary education

^e^Intermediate general secondary education/vocational education

^f^Higher general secondary education, higher vocational education, preuniversity or university level

^g^Without the Norwegian study number 9 which cannot be calculated

Frequency distribution, means, and standard deviation (SD) were calculated for the FOG and SMOG grades in the PICDs.

## Results

Through our search strategy, we retrieved 721 Dutch studies, 256 Danish studies and 192 Norwegian studies. After the screening, we included 24 studies, of which seven were Dutch, seven were Danish, and ten were Norwegian. Five of the included studies were qualitative, and 19 were quantitative. The number of participants in the studies ranged from 13 to 333. A total of 2972 participants were included in the 24 studies. In 23 of these studies, 1619 participants out of 2865 participants (57 %) had a lower educational or schooling level than the highest scores required for the FOG or the SMOG. The percentage of participants with lower educational or schooling level than the required FOG and the SMOG scores ranged from 5 % to 84 % in the included studies (Table 1). In one of the 24 studies, it was not possible to calculate the proportion of participants with a lower educational level than that suggested in the FOG/SMOG scores, because the results were presented as mean values [29] (Table 1).

Education or schooling levels presented in the included studies were often graded in two or three levels. The highest educational level was often described to be more than 12 or 13 years of schooling or as ‘university level’.

All PICDs were written in standard templates without illustrations, figures or other initiatives that could improve the readability. During the analysis, there were minor discrepancies, which were discussed. The reasons were often words that were overlooked, or that the pronunciations were unknown for one of the authors and thus the number of syllables could be interpreted differently.

The total mean score for the FOG and SMOG grades were 14.2 (9.0–19.0) and 14.2 (12–17) respectively. The mean scores for the FOG and SMOG were 12.7 and 13.3 in the Dutch studies, 15.0 and 14.9 in the Danish studies, and 14.6 and 14.3 for the Norwegian studies, respectively (Table 2).

**Table 2:** Gunning’s Fog Index (FOG) and Simple Measure of Gobbledygook (SMOG) mean scores in the PICDs in the Dutch, Danish, and Norwegian studies

	Number of values	Mean (min-max)	Std. Deviation
FOG-NL	14	12.7 (9.0–17.6)	2.6
FOG-DK	14	15.0 (11.0–18.7)	2.5
FOG-NO	20	14.5 (9.8–19.0)	2.4
SMOG-NL	7	13.3 (12.0–15.0)	1.0
SMOG-DK	7	14.9 (13.0–17.0)	1.4
SMOG-NO	10	14.3 (13.0–15.0)	0.7
FOG-All	48	14.2 (9.0–19.0)	2.6
SMOG-All	24	14.2 (12.0–17.0)	1.1

## Discussion

This study investigated whether the readability of the PICDs corresponds with the reported education or schooling level of the participants involved in rheumatological studies in the Netherlands, Denmark, and Norway.

The results show that in all 24 studies, the PICDs had a higher readability level than recommended for health literature in general [15–17], and that in all studies there were participants with a lower educational or schooling level than the required readability level in order to be able to read and understand the PICD used in the study. This corresponds with findings from a new study of 522 information sheets for research studies in the UK [30].

In total 57 % of the participants had less education or schooling than required to read and understand the PICD based on the readability scores for the FOG and SMOG. When participants have a lower level of education than the readability of the PICD, it can lead to a ‘readability gap’, meaning that participants will not fully understand the text in the PICD or what they are agreeing to [31]. This may cause participants to refuse to participate in the study or imply that they are participating in the study without giving valid consent. Participants’ understanding of the text can be improved by oral information or discussion with a study team member about the content in the PICD [22]. It is also possible to increase PICDs’ readability by using short words and phrases, headings, plenty of space, bulleted lists, illustrations, and large type [20, 32, 33].

An individual’s health literacy depends on more than education; for example, it can depend on how familiar the reader is to the health care system and the presented information [34]. When readers possess such knowledge, they may understand the text in PCIDs even when their educational level is lower than the readability level of the PICD in question. Conversely, when readers lack this knowledge, they may fail to understand the text even when they their educational level corresponds to the readability level of the PCID.

The high readability level of the PICDs in this study can possibly be explained by the regulatory requirements in national guidelines, which give more attention to the content of the PICDs than their readability. In Denmark and Norway, there are no stated requirements or focus on the level of readability of the PICDs. In the Netherlands, the ethical committees recommend to write the material on a schooling level of lower secondary school, which is approximately at an age of 11–12 years. The literature has shown that the readability level of PICDs in USA, Australia, South Africa, India and several European countries does not fit requirements so it is plausible to suggest that it is a common and worldwide problem [16, 19, 21, 35–38]. Our findings and how we have conducted our study can therefore be relevant for health professionals working with PICDs.

For all the included studies, we received the PICD from the first author, and several authors commented that the current study was an important piece of work that they would gladly support. In this study, it was difficult to find 10 Dutch and 10 Danish studies in which education or years of schooling was reported as part of the participants’ demographic data. This indicate a lack of focus on the participants’ educational of schooling level, although social inequality is an important topic.

Patients’ educational level may affect how participants manage their disease and treatment [39]. Educational level can also influence the risk of disease [40], disease severity [41], psychological distress [42], health [43], involvement in healthcare [44], and mortality [45]. Therefore, it may be advantageous for studies to report the educational or schooling level of their participants.

This study’s strength is that it examines PICDs from studies in three different European countries. It enables us to obtain knowledge on the readability of PICDs in a larger context. Using two different tests for readability also strengthens the study. Average values for FOG and SMOG in the PICDs are quite similar in our study, but within individual PICDs, there is a wide variation in scores. This shows that the complexity of PICD texts varies and that it is difficult to obtain a single readability measure of the PICDs. Readability tests like FOG and SMOG have been criticized for focusing on the document alone, and for not capturing medical terms and difficult words [12]. A weakness of a readability test is that it does not take into account the context in which the document is used [12].

For assessment of PICDs and other written patient information, there are several online tools which involve FOG, SMOG, and other readability tests. These electronic tools make it possible to quickly obtain the value of a text’s readability. Online tools are not exact and provide somewhat different results than tests scored by hand. One such tool calculates a total readability score based on several tests (http://www.readabilityformulas.com). Another tool shows the readability for each test and provides a list of which sentences should be rewritten to improve readability (http://www.online-utility.org/english/readability_test_and_improve.jsp). This tool is used in an example of PICD where the same text is written with high and low readability, see Additional file 1.

In our study, several of the PICDs were only available on paper. We also wanted to calculate the readability level as exactly as possible. Therefore, all PICDs were scored by hand.

### Study limitation

The studies included in this study use different ways of describing the participants’ level of education. Some use the number of years of schooling, grouped or as average. Other studies use low-medium-high educational classifications, and some studies describe the professions or occupations of the participants. This makes it difficult to compare results. In most studies, there will be more participants who have lower educational levels than PICDs’ readability level (Table 1), but the way participants’ education is presented in the studies makes it impossible to perform a more accurate calculation. For example, when the number of participants in a study is presented with an education level of more than 12 years and the highest readability score is 19.0, then only a few in this group will have a schooling level equivalent to 19.0 years. A standardized way of presenting participants’ educational level is needed.

The FOG and the SMOG were developed to score texts written in English, which together with Dutch, Danish, and Norwegian, belong to the Germanic language family [46]. All four languages have common language characteristics. This is why we assumed we could use the FOG and SMOG procedure in all three languages even though they are not yet validated in the three languages. The endings which are considered to be easy to understand varies between languages. We cannot ignore the fact that the scores may be influenced by the languages.

## Conclusion

More than 57 % of the participants in the included studies had a lower educational or schooling level than the readability level of the PICDs. This raises concerns about the validity of the participants’ consent. It would be relevant to develop national recommendations for the readability of PICDs. In addition, the involvement of patient research partners may help ensure a highly necessary focus on participants’ reading level. Further research is needed in other medical fields and in other countries.

### Practice implications

Our findings show that there should be more focus on the formulation and form of PICDs. The development of guidelines for writing PICDs would ensure that focus is placed on the formulation of a text that is clear and understandable. The use of such a text along with oral information will enable participants to understand a study’s contents and consequences and give valid consent. This will also ensure the best possible recruitment in the studies.

## Abbreviations

DK, Denmark; FOG, Gunning’s fog index; NL, Netherlands; NO, Norway; PICD, Patient information and consent document; SMOG, Simple measure of gobbledygook.

## Additional file

Additional file 1: PICD with high and low readability (invented). (DOCX 15 kb)
