# Supplementary material for: Readability of patient information and consent documents in rheumatological studies
Source: BMC Med Ethics. 2016 Jul 16;17:42. doi: 10.1186/s12910-016-0126-0 (PMC4947296; doi:10.1186/s12910-016-0126-0)
Supplement: Additional file 1: — PICD with high and low readability (invented). (DOCX 15 kb) [file 12910_2016_126_MOESM1_ESM.docx]

**Additional file 1: PICD with high and low readability (invented)**

**Example 1 PICD with high readability score**

Dear …

**We want to request you about participating in a scientific study about evaluation of outpatient rehabilitation and education group programs in the musculoskeletal pain department at the Hospital for Rheumatic Diseases**

**Information about the study:**

The hospital’s department of musculoskeletal pain has offered an outpatient rehabilitation and education group program since 2010. Now we want to evaluate this outpatient group program. We will therefore examine whether or not this training and education program helps increase patients' quality of life and ability to cope by conducting a randomized controlled trial. The Norwegian Research Council in collaboration with the hospital funds the study.

**What does it mean to participate in this study?**

Once you agree to participate in this study, in addition to your usual medical treatment, you will be randomly assigned to start a certain outpatient group program now or sometime within the next 12 months. The outpatient training program consists of ten group sessions of five hours each every two weeks.

The program takes place at the rheumatology department and will be led by a physiotherapist and an occupational therapist. The first day consists of an introduction to the program and a medical consultation for each attendee with a rheumatologist. During the ten group sessions, you will also meet and talk to and participate in education provided by a nurse, a social worker, a psychologist and a nutritionist.

Everyone must fill out a questionnaire on the first day of the program, as well as three and 12 months after the intervention is completed. When the survey is completed, the results will be published in reputable medical journals and on the department's website. Participants’ names will not be used in publications.

All information will be treated with confidentiality and in de-identified form. All collected data are treated with confidentiality under the Public Administration Act and the Act of Health Professionals.

**Duration of the project:**

The project will start in January 2016 and be completed by September 2018. Adaptation and publication of the data will take place within two years after the project ends. The data will be anonymized before September 2018.

The project is assessed and advised by the Regional Committee for Medical Research Ethics in Central Norway. The project has also received approval from the Patient Safety Board in the Norwegian Social Science Data Services (NSD). It is voluntary to participate in this research, and you can withdraw at any time without having to justify your reasons for doing so and without any negative consequences for your further treatment and follow-up visits to the hospital’s patient department.

If you need further information, please contact the project manager Kristine Kristoffersen. Her phone number is +47 xxxxxxxx.

In this example, the score of the FOG is 15.9 and the SMOG is 14.8

**Example 2 PICD with low readability score**

Dear …

**We want to request you about participating in a study.**

The study is about outpatient rehabilitation and education group programs.

The study will take place in the musculoskeletal pain department.

Which hospital: Hospital for Rheumatic Diseases.

**About the study:**

The hospital has an outpatient rehabilitation and education group program. We want to evaluate it. We want to see if it helps improve patients' lives. The hospital and the Norwegian Research Council paid for this survey.

**How can I participate?**

We will put you in an outpatient group program. This will happen during the next year. It has ten sessions. Each one lasts five hours. These sessions occur every two weeks.

The program takes place at the rheumatology department. A physiotherapist and an occupational therapist lead it. The first day is the introduction of the program. Next, you visit a rheumatologist. In other sessions, you will meet a nurse, social worker, psychologist and nutritionist.

On the first day, everyone answers a list of questions. Three and 12 months after the survey, you will answer more questions. We will publish the survey results in medical journals. We will also publish them on the hospitals website. We will not use your name.

All information is kept private.

**How long does the project last?**

The project starts January 2016. It ends September 2018. Within the next two years, we will analyse and publish the data. Before September 2018, we will anonymize the data.

The Regional Committee for Medical Research Ethics assessed and advised this project. The NSD Patient Safety Board approved it. It is your choice whether you want to participate. You can stop anytime. You do not need to explain why. There are no penalties.

For more information, contact the project manager, Kristine Kristoffersen. Her number: +47 xxxxxxxx.

In this example, the score of the FOG is 9.7 and the SMOG is 9.5.
